# Tracheotomy and Intubation

**Published:** 1888-05

**Authors:** W. S. Kendrick

**Affiliations:** Atlanta, Ga.


### TRACHEOTOMY AND INTUBATION.

BY W. S. KENDRICK, M. D., ATLANTA, GA.

There is scarcely a physician, having been actively engaged in the practice of medicine for a few years, but has witnessed one or more deaths from some form of laryngeal obstruction. On such an occasion the medical attendant is severely taxed to know what to do—whether he shall cease his efforts at the border line between medicinal and surgical treatment, or whether he shall resort to the latter means to attempt to save his patient. As to whether surgical measures should be resorted to or not, it can be safely said that after all medicinal means have failed, after every effort has been thwarted to give permanent relief to the sufferer, then recourse must be had to tracheotomy, intubation, or some other method, to relieve if possible the asphyxia and snatch the patient from impending death. As to tracheotomy there are various conditions that influence the operation, whether it should be done or not, but there is scarcely a doubt that it has saved many from an early and untimely grave, and many more would have been saved had a proper discrimination been made and the operation promptly executed.

Tracheotomy is one among the oldest surgical operations—in great favor at one time, in very great disfavor at another; through many centuries it has had its successive rises and falls, its strong advocates, its bitter enemies. Performed in the most infectious and malignant forms of diphtheria, it has failed to relieve because abused; again, rejected under more favorable circumstances, death has ensued when timely interference would have saved the patient. It is not our intention to criticise the acts of others, but briefly to state the conditions that demand it—some of the complications that may arise and the methods of performing it. Tracheotomy should not be resorted to until all means have been exhausted, medicinal and otherwise, for the relief of the sufferer. It should not be made until all hope from other sources has departed, until the lengthening shadows and gathering clouds plainly portray the coming storm, amid whose fury death will claim its victim. The good or bad results are usually due to the form of the disease and the time at which the operation is made.

If the disease be infectious or malignant diphtheria, making its great impression, not only on the respiratory organs, but the whole body, the operation will be much less likely to succeed than if it be simple croup limited to the upper portion of the respiratory tract. Again, if it be simple croup the prognosis would be decidedly more unfavorable if the disease has attacked the bronchi and lungs; or, whatever may be the pathological condition, if postponed until the sufferer has been largely asphyxiated, until the air has been mainly cut off from the lungs and the system is being rapidly poisoned for the want of oxygen and the presence of carbonic acid gas, of course the result would not be such as if performed earlier. For the organs to perform their functions properly and to be maintained in a healthy condition, oxygen must enter the lungs and through the blood be distributed to all the tissues of the body, and carbonic acid gas must be gathered up and eliminated from the body through the same channels.

Whatever interferes with either process will disturb very greatly the machinery of the entire body. *Tracheotomy and intubation are not operations for the cure of croup, but for the relief of the asphyxia.* They are applied entirely to the obstructions which exist in the larynx—the main cause of the asphyxia; this being removed, the proper changes take place in the pulmonic circulation, the asphyxia is gradually removed, the disease runs its course and recovery is the happy result. If a mistake should be made in the diagnosis and the disease be one of true infectious, malignant croup or diphtheria, the strong probabilities are that the patient will succumb to the general poisoning, to a renewal of the asphyxia, or an extension of the membranes down the bronchi. The ultimate success of the operation depends, to some extent, on the age of the patient. It has been performed on children only a few weeks old with good results, but children under two years do not do so well as those who are older, for the reason they are more difficult to manage, necks usually fatter and shorter, resistive forces not strong, accidents of operation much greater, tendency to eruptive fevers and more trouble to nourish.

Statistics have also proven that the operation is exceedingly fatal in adult age, the most favorable time being from three to fifteen years of age; also that success is much greater in the summer and autumn months than winter and spring, for the reason the latter months tend to develop pneumonia or broncho-pneumonia much more than the dry, warm months of summer. Broncho-pneumonia is the bane and scourge of those tracheotomized, but happily occurs much less frequently, since the cravat has been used over the mouth of tube after it is inserted and described further on. The main indication for tracheotomy is the asphyxia. Differences have existed as to the stage and intensity of this condition, and it is a nice point to decide when surgical interference shall take the place of medicinal. For convenience we would divide the disease into three stages. The first is where the patient has not reached the time of even partial asphyxia; while the breathing may be labored, the symptoms distressing, yet a sufficient amount of air enters the lungs to properly oxygenize the blood and afford the proper interchange that takes place in normal respiration. The second is where the obstruction becomes so great, whether in the larynx and trachea or further down, as to interfere so much with the functions of the lungs that the patient has moments of partial asphyxia and intervals of consciousness. The third is where the obstruction has become so great, and the system so thoroughly poisoned through failure of the lungs to perform their work properly, that the stage of complete anæsthesia is reached without any hope of returning consciousness except by surgical means. The question then comes, when shall we operate, during the first stage when no asphyxia, during the second when partial, or wait until the third when the asphyxia has gone so far as to completely anæsthetize the sufferer, when, with pallid skin, clammy sweat and labored breathing, death soon ends the scene? As a rule, all other things being equal, it is better not to wait until the third stage has been fully developed, if the patient can be seen in time, as the vital forces might be so far spent that it would be impossible to bring permanent reaction. On general principles, the operation should be made before the time of complete anæsthesia has been reached; though no stage of the disease, how much so ever profound the anæsthesia might be from the poison, should arrest the hand of the operator; he should be willing to take all the chances and risk his judgment, as the case would be necessarily fatal without it. It is not the treatment of croup, but the results that demand surgical interference.

Numerous authorities have presented strong contra-indications against tracheotomy, especially if the inflammation has extended down the bronchial tubes to the lungs, giving rise to either broncho-pneumonia or pneumonia. The former has been considered by some as positively setting aside the operation, it being the most common and dangerous of all complications. If an obstruction in the larynx above and the bronchial tubes below, what shall be done? All medicinal means have been tried and failed. Friends are anxious; the slender cord of life is at its utmost tension—two obstructions, one above, the other below. Shall the surgeon stand with folded hands a quiet spectator, an unmoved looker-on in this scene of misery and death, or shall he act, and act promptly, by opening the windpipe below one obstruction and thus relieve the sufferer of that much of the trouble? It seems that would be the safer and better plan. Many cases of true membranous or diphtheritic croup will die if operated on at any stage; yet there should be but one positive objection, and that is when the larynx is entirely free and the obstruction is lower down the respiratory tract and cannot be reached; but if confronted with a true axphyxia, with stenosis of the larynx, though surrounded by grave complications, it becomes our duty to resort to tracheotomy or intubation. The different methods of making the operation are familiar to those who have given the subject special thought. A few words will no doubt suffice as to the way it should be made. If possible, skilled assistants, with all the necessary surgical appliances, should be on hand, though it sometimes happens that the case is so urgent that neither can be had, and the surgeon has to hastily improvise such instruments and means with which to act, having no time to wait. Daylight is always preferable to night if it could be arranged. Instruments required are not numerous: bistoury, forceps, blunt hooks, dilators, double-curved canulas, and any others the operator may think necessary, are the main ones. A canula as large as the larynx or trachea will admit, so that respiration may be freely performed, is the best for use. Besides water, sponges, tape, etc., a piece each of tarlatan and flannel, to make a cravat to cover the mouth of the canula after it has been inserted, should be on hand. Complications, including broncho-pneumonia, have not been so frequent since the introduction of the cravat. The reasons are obvious: In normal respiration the air passes through the nose and pharynx where it is warmed, moistened and relieved of floating particles of dust, etc., before coming in contact with the delicate membranes of the bronchial tubes and air vesicles. Without the cravat the air would pass directly into the lungs, neither warmed nor moistened, and loaded with its extraneous substances, while the presence of the cravat tends to supply the normal respiration through the nasal passages, thereby rendering the tendency to broncho-pneumonia much less than formerly. It is also claimed that bacteria are much more easily prevented from entering the air passages since the introduction of the cravat. If the stage of complete anæsthesia has been reached from the poison, anæsthetics may not be necessary; but as a certain amount of spasm frequently exists in the larynx, patients usually do very well with some, as it relieves the spasm, but it should be handled with extreme care. The operation may be made anywhere from the cricoid cartillage to the seventh or eighth rings. The low operation, viz., between the third and seventh rings, is attended with more danger than those higher up; the close proximity to the large vessels and the depth of the windpipe make it both more difficult and dangerous than either of the others. It is safer to cut through the upper two or three tracheal rings, or through the cricoid and as many rings as are required to make the incision long enough, besides the upper portion is nearer the surface than the lower. Different operators advocate different methods, but whichever is selected the cut should be made immediately in the median line so as to avoid as much hemorrhage as possible and to properly enter the windpipe. Three steps begin and complete the procedure, viz., division of soft parts, opening trachea and introducing canula.

Presuming the anatomy is well known, we will not go into detail as regards every step of the operation. Whatever landmarks can be mapped out, that will enable us to keep in the median line immediately over the trachea until it has been reached, will be sufficient, great care being taken to use the utmost precautions as to wounding important vessels and cutting through both vessels and tissues that might have been avoided. When the trachea has been reached, the bistoury should be entered and a sufficient cut made, up or down, as the case may be, to admit a canula that will meet all the indications—that will allow a sufficient amount of air to enter the lungs. It is alway safest and best to arrest all hemorrhage from the soft parts before the trachea is opened, as the blood might enter the windpipe and further complicate the case. A sharp, hissing sound made by the air entering usually indicates that the passages have been opened. The next step is the introduction of the canula, and it is at this point the surgeon should maintain his coolness and dexterity. Either with a finger-nail or dilator in the edge of the wound the canula is introduced. As soon as the wound is separated there usually comes a shower of blood, false membranes and mucus flying in all directions; but if the canula is properly introduced, and the cause of the asphyxia above the wound, good results almost immediately follow. As soon as the canula is in proper place it should be secured with tape and the cravat of woolen and tarlatan tied over it and the patient put to bed. Whether the operation should be made slowly or rapidly is a question for the exigencies of the case to decide. If a patient is in immediate danger of death, it should be done at once and done rapidly; if, on the other hand, death is not so imminent, there will be no great necessity for such hurry. If the case progresses well, the canula should not be removed for twenty-four hours, at which time it should be properly cleaned and put back. This change should be made every day until the patient is entirely relieved. When the air begins to pass through the larynx with sufficient ease, the instrument should be allowed to remain out a few moments each day until it can be finally withdrawn. As soon as the patient can breathe free and easy without labored effort, and the sputa from the trachea passes through the mouth, its final removal is indicated. The wound should be properly dressed, well protected and left to heal. A few days only may be necessary, or it may be quite a while before the process is complete. That will depend on the general condition of the patient and the complications that may arise. The patient should be carefully nursed and fed, and all indications must be met and handled as the case may demand.

A more recent method for the relief of laryngeal obstruction is intubation. This plan has recently been revived by Dr. O'Dwyer, of New York, with improved instruments for its accomplishment, and bids fair, if statistics are correct, of becoming a formidable rival for those claiming greater advantages for tracheotomy. It is probably much less dangerous, more easily performed and meets with the approval of the family and friends of the patient more frequently than the operation described above. It has its advantages and disadvantages, but it is at least possible that it will largely supplant tracheotomy in the future. We cannot go into a minute detail in describing the instruments used and the manipulations necessary, but will briefly say they consist in placing a tube in the larynx through the mouth, which, by its make, is readily held in position by the larynx, and which enables the patient, if there is no obstruction lower down, to supply the needed air to the lungs. As a rule, the tube can be worn for days without any great inconvenience, and inserted and removed without very great difficulty. If a tube can be properly introduced and well borne by the patient, it offers the means to get air into the lungs without the unpleasantness and risks of such an operation as tracheotomy. The future only can decide by correct statistics whether intubation is a mere novice in the profession, or whether it shall take high rank with other surgical means for laryngeal and tracheal stenosis.

